# The effect of APN, hs-CRP and APN/hs-CRP in periodontitis with DAA

**DOI:** 10.1186/s12903-023-02765-x

**Published:** 2023-02-10

**Authors:** Rui Cheng, Xiaojiang Xu, Shurong Yang, Zhongqian mi, Yong Zhao, Chong Wang, Xuexue Shi, Jinhua Gao, Feiyan Yu, Xiuyun Ren

**Affiliations:** 1 Department of Endocrinology, The Second Hospital of Shanxi Medical University, 382 Wuyi Road, Taiyuan, 030001 Shanxi Province China; 2 Shanxi Medical University, 56 Xinjian South Road, Taiyuan, 030001 Shanxi Province China; 3 Shanxi Medical University School and Hospital of Stomatology, 63# Xinjian South Road, Taiyuan, 030001 Shanxi Province People’s Republic of China; 4 Shanxi Province Key Laboratory of Oral Diseases Prevention and New Materials, Taiyuan, 030001 China

**Keywords:** Chronic periodontitis, DAA, APN, Hs-CRP, Lipid

## Abstract

**Background:**

Common chronic infections induced low-grade inflammation has been correlated with atherosclerosis as supported by strong evidence. The balance between pro-and anti-inflammatory factors was exploited to elucidate the effects of chronic periodontitis on diabetes-associated atherosclerosis.

**Methods:**

Study subjects encompassed 30 SPF male rats randomly divided into four groups: A group (NC), B group (T2DM), C group (CP), D group (DM + CP). After developing the model, blood samples were collected from the angular vein analyze serum APN, hs-CRP, and blood lipid. the carotid artery was isolated for HE staining.

**Result:**

Compared with group A, the serum APN in group B, C and D decreased gradually with the progression of the disease. Serum hs-CRP in group B, C and D was significantly increased. At T3, T4 and T5 in group B, C and D, APN/hs-CRP significantly decreased. TC, LDL and TG significantly increased in group B, D; HDL significantly decreased in group C. Carotid artery HE staining showed: compared with group A, different degrees of endothelial defect, destruction of elastic fibers in the middle membrane, disorder of smooth muscle arrangement, and partial dissolution 、 fragmentation and Calcium salt deposition necrosis occurred in group B, C and D.

**Conclusion:**

Enhanced systemic inflammation, decreased adiponectin level, and disorganized lipid metabolism with or without type 2 diabetes attributed to local inflammation of periodontitis can result in an imbalance of pro-inflammatory and anti-inflammatory effects. Therefore, it’s more meaningful to predict the progression of DAA with anti-inflammatory/pro-inflammatory variation.

**Supplementary Information:**

The online version contains supplementary material available at 10.1186/s12903-023-02765-x.

## Introduction

Macrovascular complications, as one of the major complications of Type 2 Diabetes Mellitus (T2DM), are the primary cause of disability, with a mortality rate of 65–75% [1]. Strong evidence substantiates the association of low-grade inflammation with many chronic diseases, for example, obesity, diabetes mellitus, atherosclerosis, and chronic periodontitis [2–4]. The incidence of periodontitis is as high as 60–70% in China. Periodontitis is a multifactorial, chronic inflammatory disorder with non-reversible damage to tooth-supporting tissues (gingiva, periodontal ligament, alveolar bone), leading to tooth mobility, tooth loss, and concomitant effects on oral function and life quality [5]. The continuous low-grade infection allows the microorganisms and virulence factors to enter the circulation and lead to systemic inflammation [6] and therefore, periodontitis is regarded as a risk factor for diabetes mellitus, cardiovascular, and cerebrovascular diseases [7–10]. Many evidence suggests that both T2DM and AS are chronic inflammatory diseases and are driven by inflammation [11, 12].

Adiponectin/APN, also known as ACRP30 (Adipocyte complementary related 30Kda protein), is a specific fatty molecule secreted by adipose tissue [13]. Adiponectin exerts its biological role by binding to seven transmembrane receptors, adiponectin receptors 1 and 2 (AdipoR1 and AdipoR2), which are coupled to two structurally and functionally different G proteins [14]. Reduced expression of adiponectin and its cell surface receptors are linked with obesity, obesity-related insulin resistance, and chronic inflammatory status of diabetes [15]. Clinical studies confirmed the association of atherosclerosis with adiponectin [16]. A negative correlation of APN with inflammatory cytokine is attributed to the inhibition of TNF-α stimulated IL-8 synthesis in endothelial cells by regulating the NF-κB signaling pathway [17]. Wei Qiu et al. [18] found that APN receptor agonist AdipoAI impedes lipopolysaccharide-induced endotoxemia and other inflammatory diseases by triggering different signaling pathways.

The acute-phase reactant C-reactive protein (CRP) has long been considered a useful marker of inflammation. The vital role of CRP is also noted in host defense against invading pathogens and inflammation. Many studies have highlighted CRP as an independent cardiovascular predisposing factor, independent of high cholesterol and LDL [19]. Moreover, the prognosis of cardiovascular disease can be improved by reducing CRP levels [19].

Periodontitis is a common and highly frequent local inflammatory disease. Nonetheless, the mechanism of promoting diabetes-related atherosclerosis is ambiguous. Studies have substantiated the close relation of both APN and PCR to the progression and prognosis of atherosclerosis. However, there are limited studies that address the role of the balance between the anti-inflammatory effects of APN and the pro-inflammatory effects of CRP in atherosclerosis.

## Materials and methods

### Animals

Twenty-eight 6-week-old SD male rats weighing 180–200 g were purchased from the animal center of Shanxi medical university. All rats were maintained under standard housing conditions following the protocol approved by the Institutional Committee for Animal Use and Care at Shanxi medical university (No.2018LL346). Arrive Statements: (i) identifying the institutional and/or licensing committee approving the experiments, including any relevant details; (ii) confirming that all experiments were performed in accordance with relevant guidelines and regulations. All rats were randomly divided into the following groups: control rats (A group n = 7), T2DM rats (B group n = 7), periodontitis rats (C group n = 7), T2DM + CP rats (D group n = 7).

### Modeling

**Fig. 1:**
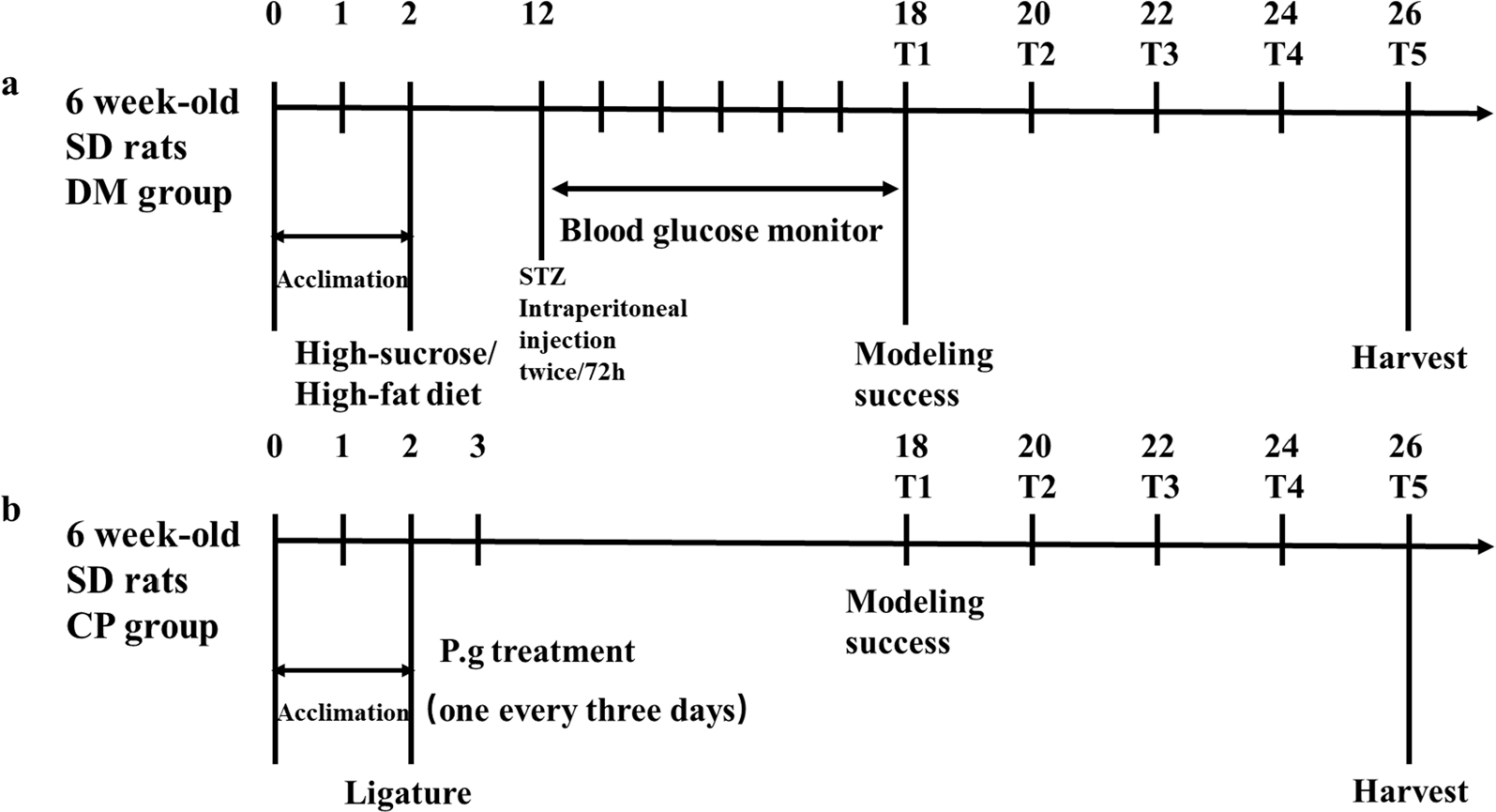
Timeline of this work. **a, b** The experimental time schedules of the diabetes mellitus (DM) group and chronic periodontitis (CP) group, respectively

T2DM: at 8 weeks of age, rats in the B group and D group were provided Western diet (Homemade: 20% sucrose + 10% lard + 2.5% cholesterol + 1% pig bile salt + 66.5% basal feed), multiple low doses of streptozotocin(STZ, Sigma–Aldrich, USA) (25 mg/kg) were injected intraperitoneally, and dynamic monitoring of blood sugar, Model standard: FPG > 7.8 mmol/L, or PBG ≥ 17.8 mmol/L (Fig. 1a).

CP: the experimental teeth (bilateral maxillary first and second molar) were ligated under general anesthesia with Oral ligation wire and 3–0 silk thread. A week later, P. g suspension was applied once every 3 days and checked regularly. The bacteria were applied a total of 15 ~ 20 times. The standard strain of Porphyromonas gingivalis (Pg) was provided by the Laboratory of Oral Microbiology of Capital Medical University (ATCC33277), which was mixed to a concentration of 1 10^6^ CFU mL^–1^ and used on the same day (Fig. 1b).

T2DM + CP: the periodontitis model of rats was established at the early stage of the Western diet. After developing the model successfully, the rats continued to be fed.

Explanations and Instructions: T1, T2, T3, T4, T5:

The experiment was divided into: control, CP, DM, CP + DM, of this groups When the modeling is successful, T1 (1 week before the first intervention), T2 (1 week after the first intervention), T3 (1 week after the second intervention), T4 (3 weeks after the second intervention), T5 (5 weeks after the second intervention), These time nodes are the time points of intervention for Group CP, DM + CP, the groups covered in this article are natural progression groups, another part of the study was to compare the natural progression at each time node with that after the intervention.

### Detection indicators and methods

After inducing the models successfully, the inflammatory index hs-CRP (ELISA kit, Shanghai Xitang Co., LTD.), adipic factor APN (ELISA kit,Shanghai Xitang Co., LTD.), the collected serum samples to be tested were removed from − 80 °C and returned to room temperature. 1:5 dilution of specimen dilution was used during the experiment (20ul sample was taken and 80ul specimen dilution was added) and blood lipids: TC (TC Assay Kit, Di Rui Medical Technology Co., LTD, CHOD—PAP, sample size: 3ul), LDL (Direct—surfactant removal method, sample size: 4ul),TG (Direct—catalase clearance method, sample size: 3 µl), HDL (GPO-PAP, sample size: 4 µl), were measured at different time points in the natural process. Finally, the rats were sacrificed (at 26 weeks) (anesthesia of animals is Pelltobarbitalum Natricum through intraperitoneal injection anesthetization) to isolate bilateral carotid arteries. The vascular tissues of about 1 cm above and below the bifurcation of bilateral carotid arteries of rats were separated. After soaking in normal saline, the left side was placed in neutral formalin solution and fixed for 24–48 h. The left side was embedded in paraffin wax and cross-sectional sections were performed with thickness of 4 μm, two slices were cut continuously from each slide and observed under light microscope after HE staining.

### Statistical analysis

All the data were represented by the figures. Data were presented as mean ± SD. SPSS software (version 21) was adopted for the statistical analysis. To determine the significance, one-way ANOVA with Tukey’s post-hoc test was employed for multi-group comparisons. Statistical significance was set at *p* < 0.05.

## Result

### Establishment of T2DM model and the loss of alveolar bone

**Fig. 2:**
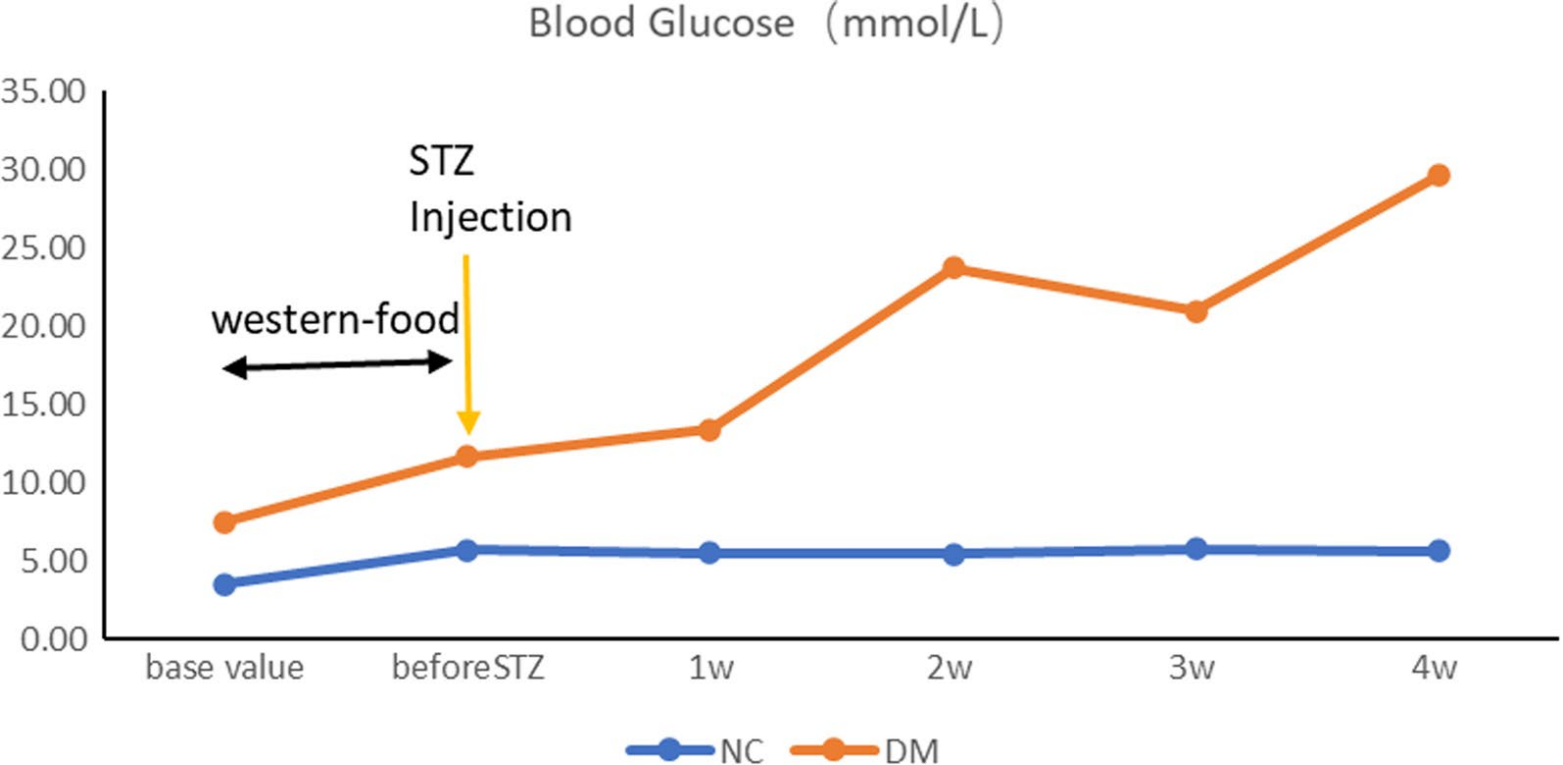
Fasting plasma glucose or random blood glucose was monitored. Model standard: FPG > 7.8 mmol/L, or PBG ≥ 17.8 mmol/L

Following two weeks of adaptive feeding, SD rats were fed with Western food for 4–6 weeks, and thereafter, multiple intraperitoneal injections of low-dose STZ (25 mg/kg) were administered, and fasting plasma glucose or random blood glucose was monitored. Model standard: FPG > 7.8 mmol/L, or RBG ≥ 17.8 mmol/L (Fig. 2).

**Fig. 3:**
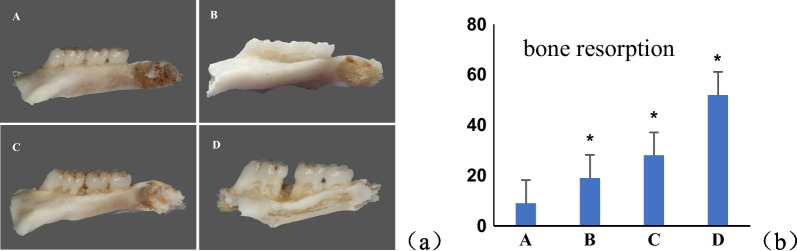
Bone resorption values of alveolar bone in each group. The CEJ value of alveolar bone in group B, C and D was significantly increased. **a** Normal control; **b** DM; **c** chronic periodontitis; **d** DM + CP, compared to group A, *p < 0.05.

The distances from the cement-enamel junction (CEJ) to the alveolar bone crest of the tooth were measured at three points (mesial, medial, and distal aspects), both in the buccal and lingual sides. The measurements of each point were repeated three times, and the mean was computed. The mean crystal bone level of the tooth was calculated (Fig. 3a, b).

### Effect of adiponectin, hs-CRP on periodontitis with or without type 2 diabetes in rats

**Fig. 4:**
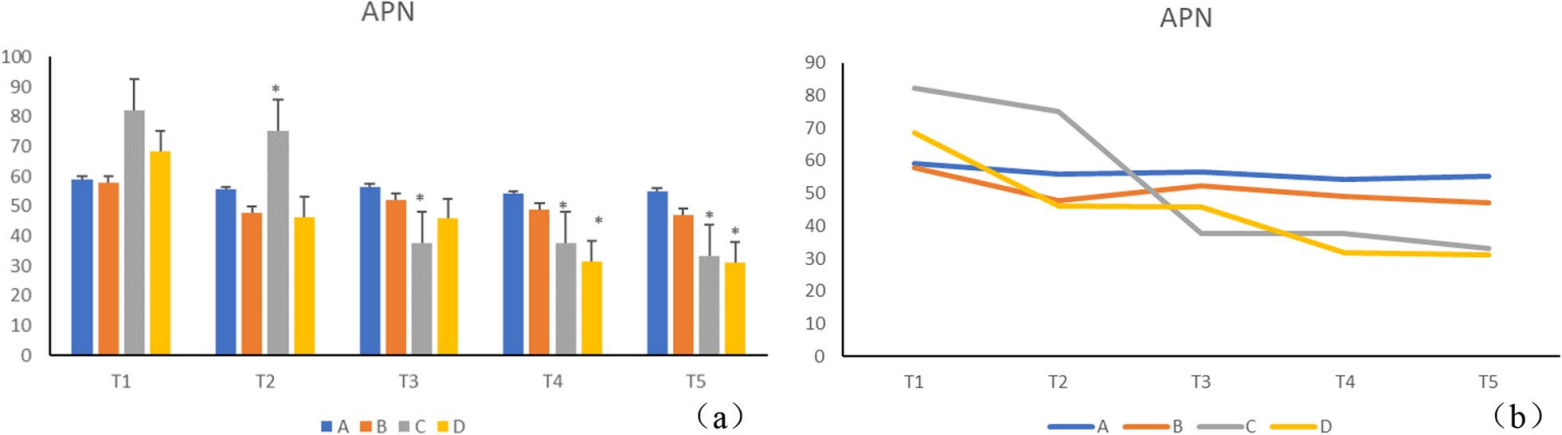
Disease progression documented a gradual reduction in the level of APN in groups B, C, and D. Serum adiponectin levels in group B decreased gradually with the progression of the disease, though the difference was statistically insignificant (*p* > 0.05). Compared to the A group, the significantly higher (*p* < 0.05)

**Fig. 5:**
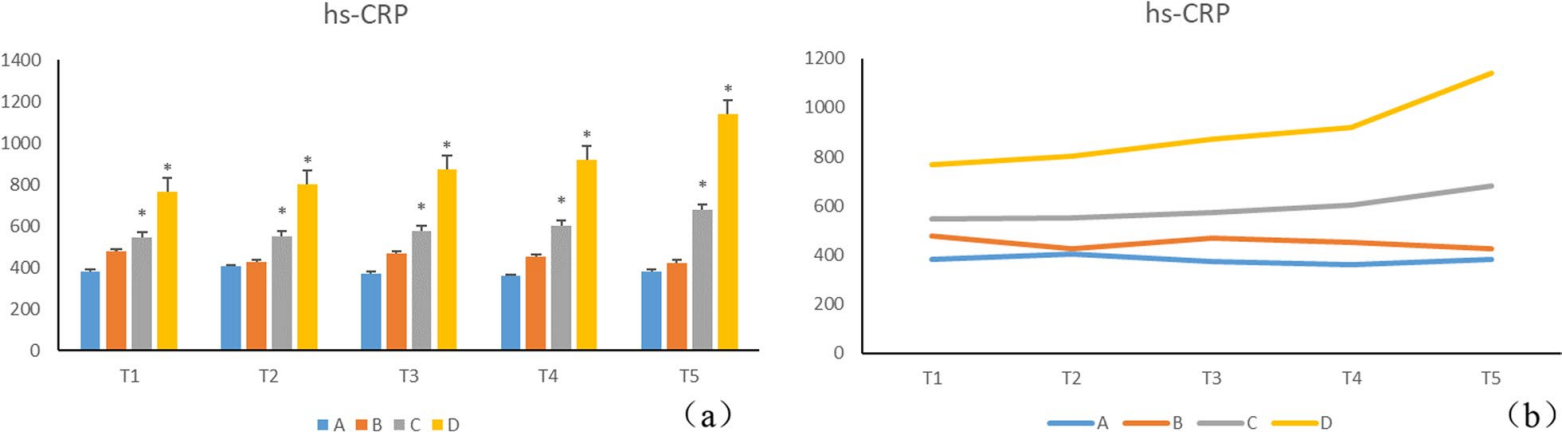
The inflammatory factor hs-CRP in natural process was higher in groups B, C, and D, relative to group A. Serum hs-CRP gradually increased with the natural progression of the disease, but group B manifested no statistical difference (*p* > 0.05)

Disease progression documented a gradual reduction in the level of APN in groups B, C, and D. Serum adiponectin levels in group B decreased gradually with the progression of the disease, though the difference was statistically insignificant (*p* > 0.05). Compared to the A group, the significantly higher (*p* < 0.05) APN level in group C at T1, T2, may be related to acute inflammation in the early modeling of periodontitis. On the contrary, at T3, T4, T5, the level of APN was significantly lower in groups C and D (*p* < 0.05) compared to group A (Table 1, Fig. 4a, b). The inflammatory factor hs-CRP in natural process was higher in groups B, C, and D, relative to group A. Serum hs-CRP gradually increased with the natural progression of the disease, but group B manifested no statistical difference (*p* > 0.05) (Table 2, Fig. 5a, b).

**Table 1:** Serum APN values of each group during natural process (n = 7, $\overline{x}$ ± s, unit: ng/ml)

Group	T1	T2	T3	T4	T5
A	59.085 ± 9.731	55.800 ± 10.498	56.586 ± 7.815	54.329 ± 5.915	55.129 ± 7.319
B	57.957 ± 13.696	47.857 ± 10.195	52.157 ± 4.433	48.985 ± 3.831	47.157 ± 1.988
C	82.071 ± 15.730	75.129 ± 6.176^*^	37.800 ± 4.699^*^	37.629 ± 3.375^*^	33.271 ± 6.205^*^
D	68.486 ± 18.038	46.243 ± 8.234	45.829 ± 8.907	31.657 ± 8.372^*^	31.143 ± 5.482^*^

A: normal control, B: DM, C: chronic periodontitis, D: DM + CP, compared to group A, *p < 0.05

**Table 2:** Serum hs-CRP values of each group during natural process (n = 7, $\overline{x}$ ± s, unit: ng/ml)

Group	T1	T2	T3	T4	T5
A	381.372 ± 33.495	403.996 ± 25.396	371.362 ± 68.992	359.266 ± 66.649	381.622 ± 63.395
B	476.051 ± 28.914	426.301 ± 32.647	468.340 ± 17.227	450.58 ± 70.09	423.347 ± 62.691
C	544.541 ± 139.025*	551.477 ± 77.024*	573.838 ± 97.880*	602.447 ± 46.125*	679.076 ± 103.723*
D	766.103 ± 91.914*	800.349 ± 97.084*	873.435 ± 96.394*	920.534 ± 176.731*	1141.06 ± 270.507*

Compared to group A, *p < 0.05

### The ratio of APN/hs-CRP in each group

**Fig. 6:**
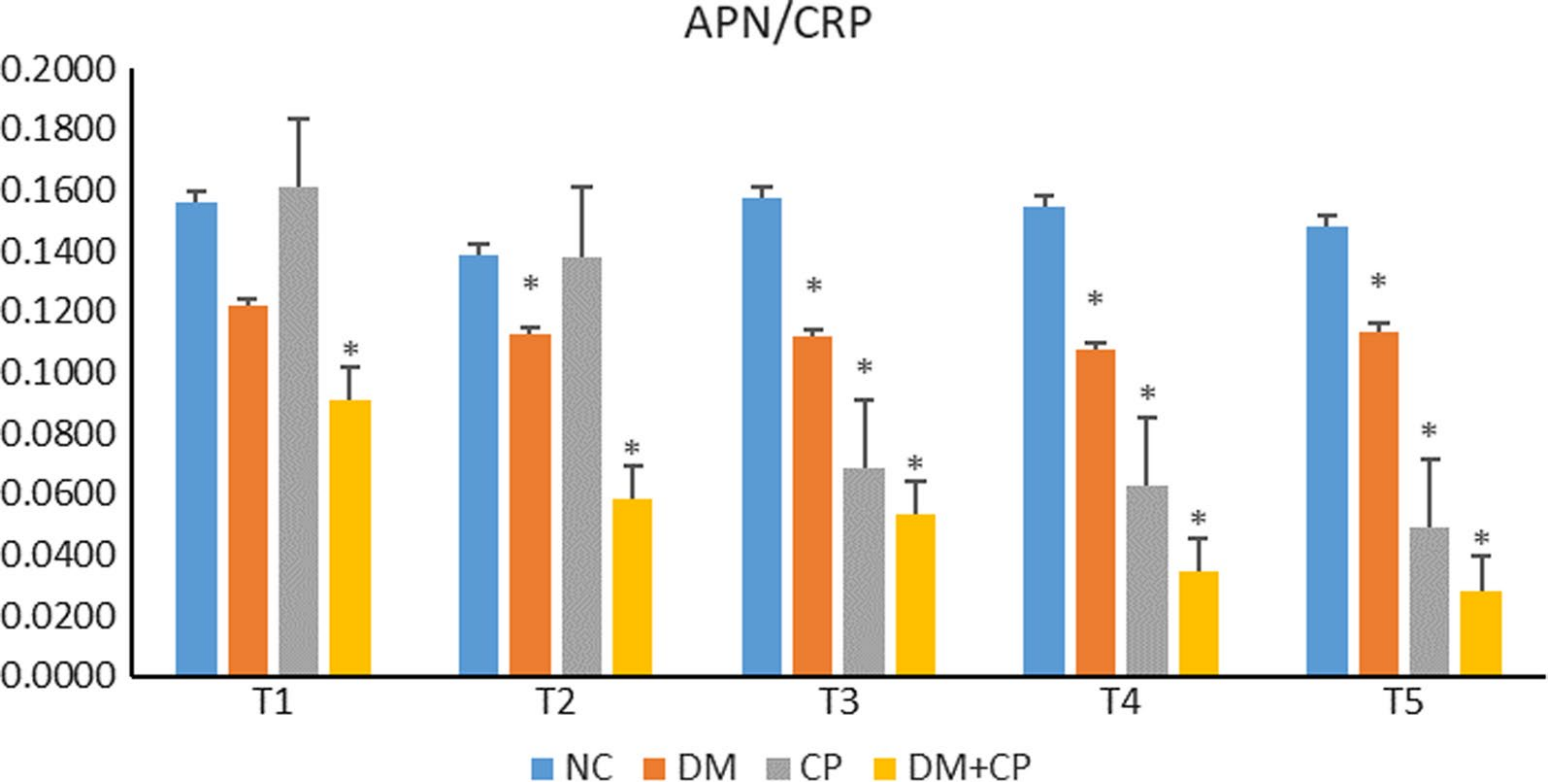
The ratio of APN/hs-CRP signified the balance between anti-inflammation and pro-inflammation. The results revealed, at T1, significantly lower (*p* < 0.05) ratio of APN/CRP in D group, but no significant difference (*p* > 0.05) in group B, C group as compared to A group; at T2, compared to A group, the ratio was significantly lower (*p* < 0.05) in both B and D group. However, C group demonstrated no difference (*p* > 0.05); the ratio was significantly lower (*p* < 0.05) in the B, C, D group at T3, T4, T5, compared to A group. Overall, with the disease progression, the ratio of the B group failed to reflect any significant downward trend, whereas, C, D group manifested a gradual downward trend

The ratio of APN/hs-CRP signified the balance between anti-inflammation and pro-inflammation. The results revealed, at T1, significantly lower (*p* < 0.05) ratio of APN/CRP in D group, but no significant difference (*p* > 0.05) in group B, C group as compared to A group; at T2, compared to A group, the ratio was significantly lower (c 0.05) in both B and D group. However, C group demonstrated no difference (*p* > 0.05); the ratio was significantly lower (*p* < 0.05) in the B, C, D group at T3, T4, T5, compared to A group. Overall, with the disease progression, the ratio of the B group failed to reflect any significant downward trend, whereas, C, D group manifested a gradual downward trend (Table 3; Fig. 6).

**Table 3:** The ratio of serum APN/CRP in each group (n = 7, $\overline{x}$ ± s)

	A	B	C	D
T1	0.1562 ± 0.0313	0.1221 ± 0.0302	0.1613 ± 0.0582	0.0909 ± 0.0280^*^
T2	0.1385 ± 0.0275	0.1125 ± 0.0242^*^	0.1382 ± 0.0205	0.0584 ± 0.0115^*^
T3	0.1577 ± 0.0412	0.1116 ± 0.0117^*^	0.0683 ± 0.0174^*^	0.0532 ± 0.0128^*^
T4	0.1547 ± 0.0257	0.1074 ± 0.0084^*^	0.0628 ± 0.0089^*^	0.0348 ± 0.0096^*^
T5	0.1481 ± 0.0316	0.1136 ± 0.0175^*^	0.0488 ± 0.0048^*^	0.0284 ± 0.0070^*^

Compared to group A, *p < 0.05

### Serum lipid profile analysis

**Fig. 7:**
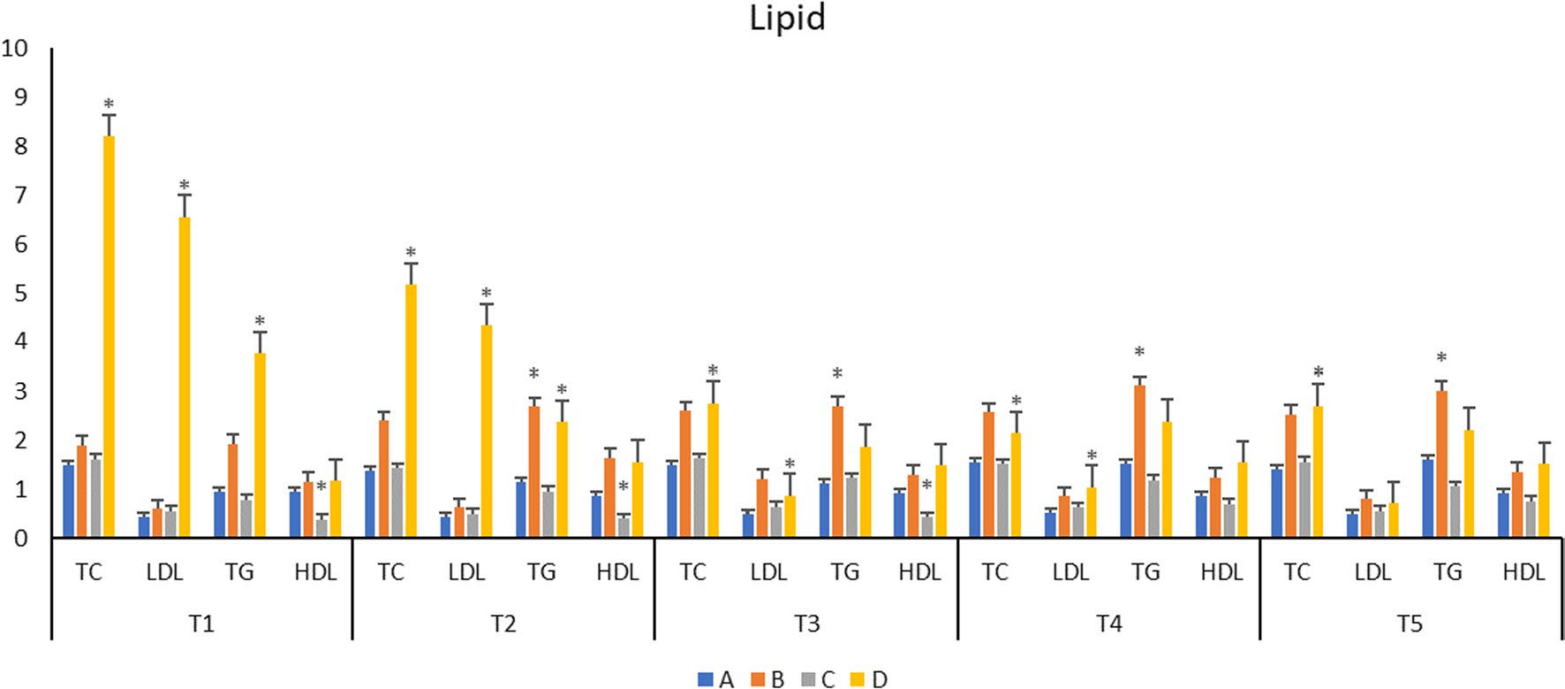
Serum lipid profile analysis. Compared to the A group, TG of group B, TC, LDL, TG of group D were significantly increased (*p* < 0.05). In the case of the C group, a significant decrease (*p* < 0.05) was obtained for HDL as compared to the A group. However, the other lipid indicators showed no significant difference (*p* > 0.05)

Disorders of lipid metabolism can induce the development of atherosclerosis. Our results revealed that compared to the A group, TG of group B, TC, LDL, TG of group D were significantly increased (*p* < 0.05). In the case of the C group, a significant decrease (*p* < 0.05) was obtained for HDL as compared to the A group. However, the other lipid indicators showed no significant difference (*p* > 0.05) (Table 4, Fig. 7).

**Table 4:** lipid values of each group during natural process (n = 7, $\overline{x}$ ± s, unit: ng/ml)

Group	T1				T2				T3			
	TC	LDL	TG	HDL	TC	LDL	TG	HDL	TC	LDL	TG	HDL
A	1.50 ± 0.26	0.45 ± 0.09	0.95 ± 0.35	0.96 ± 0.066	1.38 ± 0.26	0.44 ± 0.10	1.16 ± 0.61	0.87 ± 0.06	1.50 ± 0.22	0.50 ± 0.12	1.12 ± 0.36	0.92 ± 0.10
B	1.91 ± 0.2	0.61 ± 0.19	1.94 ± 1.06	1.16 ± 0.18	2.42 ± 0.38	0.63 ± 0.21	2.7 ± 1.09*	1.65 ± 0.33	2.62 ± 0.92	1.23 ± 0.73	2.71 ± 1.99*	1.31 ± 0.27
C	1.63 ± 0.63	0.57 ± 0.055	0.79 ± 0.146	0.40 ± 0.078*	1.44 ± 0.16	0.51 ± 0.032	0.97 ± 0.16	0.41 ± 0.06*	1.64 ± 0.21	0.65 ± 0.06	1.24 ± 0.19	0.43 ± 0.047*
D	8.2 ± 4.26*	6.55 ± 3.29*	3.76 ± 1.82*	1.19 ± 0.26	5.17 ± 3.28*	4.33 ± 3.97*	2.38 ± 1.56*	1.57 ± 0.28	2.77 ± 0.53*	0.88 ± 0.29*	1.88 ± 1.13	1.49 ± 0.26

Group	T4				T5			
	TC	LDL	TG	HDL	TC	LDL	TG	HDL
A	1.55 ± 0.19	0.5225 ± 0.06	1.52 ± 0.53	0.87 ± 0.075	1.425 ± 0.15	0.505 ± 0.05	1.625 ± 0.55	0.92 ± 0.07
B	2.58 ± 0.76	0.87 ± 0.34	3.13 ± 1.40*	1.26 ± 0.32	2.54 ± 0.64	0.81 ± 0.42	3.03 ± 1.7*	1.37 ± 0.43
C	1.53 ± 0.15	0.63 ± 0.06	1.19 ± 0.27	0.71 ± 0.057	1.57 ± 0.15	0.56 ± 0.024	1.07 ± 0.12	0.77 ± 0.058
D	2.15 ± 0.38	1.05 ± 0.23*	2.4 ± 0.77	1.55 ± 0.37	2.72 ± 0.38*	0.73 ± 0.23	2.23 ± 0.69	1.52 ± 0.19

Compared to group A, *p < 0.05

### Effects of periodontitis and diabetes on carotid artery in rats

**Fig. 8:**
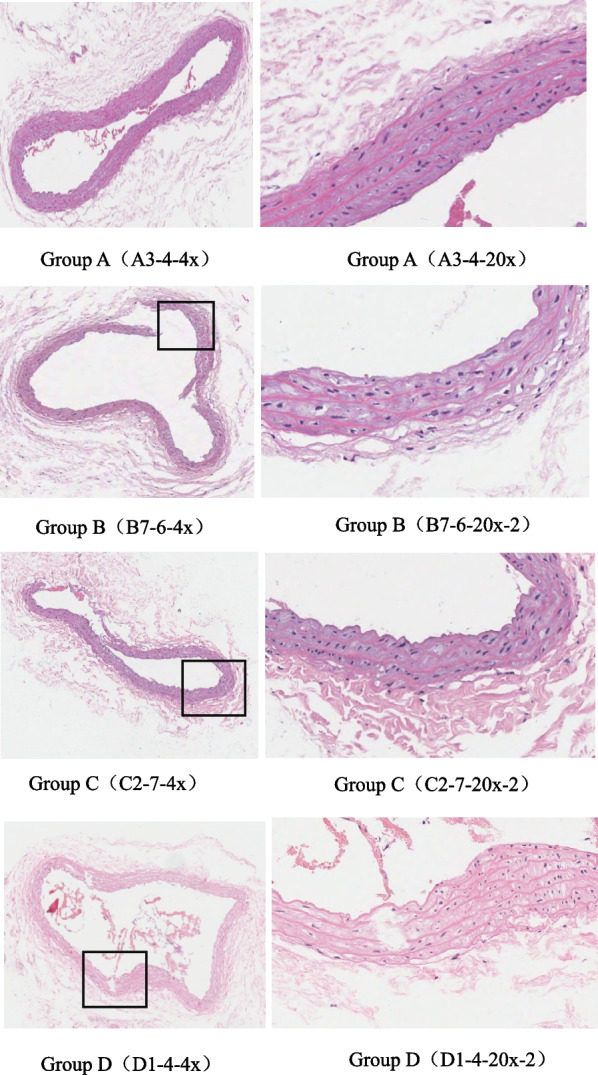
HE staining results of carotid arteries in each group. A group: an intact intima, flattened endothelial cells, orderly arrangement of the elastic fibers and smooth muscle cells of the media, no thickening of the vessel wall were observed; B group: variation was noted in the blood vessel walls thickness, part of the endothelial cells were missing, the elastic fibers of the media were disordered, partially dissolved and fractured, and the smooth muscle cells were vacuolated; C group: the intima was incomplete, and some endothelial cells were exfoliated, the elastic fibers of the media were disordered, some of them were broken, and some specimens manifested calcium salt deposition; D group: the intima was incomplete, and some endothelial cells were missing, local necrosis of the smooth muscle tissue of the media was prominent, with amorphous particles and deepened staining, the elastic fibers in the necrotic area were disordered and some of them were broken

A group: an intact intima, flattened endothelial cells, orderly arrangement of the elastic fibers and smooth muscle cells of the media, no thickening of the vessel wall were observed; B group: variation was noted in the blood vessel walls thickness, part of the endothelial cells were missing, the elastic fibers of the media were disordered, partially dissolved and fractured, and the smooth muscle cells were vacuolated; C group: the intima was incomplete, and some endothelial cells were exfoliated, the elastic fibers of the media were disordered, some of them were broken, and some specimens manifested calcium salt deposition; D group: the intima was incomplete, and some endothelial cells were missing, local necrosis of the smooth muscle tissue of the media was prominent, with amorphous particles and deepened staining, the elastic fibers in the necrotic area were disordered and some of them were broken (Fig. 8). These results suggest that periodontitis accelerates the lesion of carotid artery in diabetic rats.

## Discussion

Our results reported a reduction in the serum APN of B, C, and D groups, especially a significant decrease in the C and D groups in comparison to group A. At the same time, the pathological changes of the carotid artery were witnessed in B, C, and D group, in particular, some endothelial cells in the carotid artery of the D group were shed and lost, local necrosis was evident in the smooth muscle tissue of the media, with amorphous particles and deepened staining, the elastic fibers in the necrotic area were disordered and some of them were broken. These results implied a negative correlation of chronic inflammatory disease with serum APN level, promoting the pathological alterations of atherosclerosis, but successful modeling of periodontitis in rats reflected a significant increase in the serum APN level, however, this significant increase was only seen in T2 and APN was significantly reduced thereafter.

Adiponectin is an endogenous bioactive protein produced by adipose, a specific fat molecule with anti-atherosclerotic, anti-inflammatory, and lipid regulatory properties. And periodontitis is a inflammatory disease and is also the sixth complication of diabetes. Uncontrolled periodontal inflammation gradually destructs the periodontal supporting apparatus and leads to the consequent loss of teeth. Recently, emerging evidence has revealed an association between APN and periodontitis. Iwayama et al. study demonstrate that adiponectin exerts anti-inflammatory effects on (human gingival fibroblasts, HGFs) and (mouse gingival fibroblasts, MGFs), and promotes the activities of osteoblastogenesis of(human periodontal ligament, HPDL) cells [20]. Xingwen Wu,et al. research results demonstrate that AdipoAI ameliorates the severity of T2D-associated periodontitis by enhancing autophagy in osteoclasts at lower doses [21]. As mentioned above, the underlying mechanisms may include the functions of APN in suppressing inflammation and promoting bone regeneration [22].

Other studies have shown that adiponectin contributes to the protection of endothelial cells [23]. Our study claimed a lower level of serum APN in the B group than that of the A group, which was further substantiated by the significant pathological changes of the carotid artery. Chen, Tao, et al. observed a significant decline (*p* < 0.001) in the serum APN level of type 2 diabetic patients with the macrovascular disease. Moreover, the prevalence of diabetic macrovascular disease gradually increased with the decrease of adiponectin [24]. Another meta-analysis documented a strong association of the elevated levels of inflammatory cytokines (IL-1β, Il-6, IL-18, CRP), TNF-α, and low levels of adiponectin with the risk of T2DM [25]. Philip M. Preshaw et al. [26] found that diabetes and periodontitis can simultaneously aggravate systemic inflammation, and the decrease of serum APN in patients was more obvious with diabetes combined with periodontitis. These reports were thus in agreement with our findings. Nonetheless, the value of serum APN was significantly elevated in the early modeling of periodontitis in our study. However, at present, there is limited research on APN secretion in the initial or acute stage of inflammation. There was a study of adiponectin in the early stages of acute systemic inflammation [27]. The results claimed that the plasma adiponectin level failed to alter following vaccination, but decreased after open-heart surgery, though there was no significant change in adiponectin gene expression in the omentum and subcutaneous [28]. However, adiponectin dysregulation has been reported in another associated systemic autoimmune rheumatic disease (SARDs) [29]. It is observed in SARDs with a high inflammatory component, such as rheumatoid arthritis and systemic lupus erythematosus, where APN serum levels are elevated [30, 31]. Circulating adiponectin is related to the imaging progression of rheumatoid arthritis [32]. A positive association of the cardiovascular risk of SLE with lupus nephritis has also been reported [33]. Nevertheless, in non-inflammatory SARDs, such as systemic sclerosis, APN levels are lower and negatively correlated with disease activity [34]. The accumulated evidence supports that APN is negatively associated with chronic inflammatory disease, but studies on the early secretion of acute inflammation have not been unified. Thus, further research needs to focus on this area.

Our study reported, increased the inflammatory factor hs-CRP was increased in B group, C group, and D groups, especially in C and D groups. Corresponding carotid pathologic changes also indicated the D group was the most severe. The levels of inflammatory cytokines were consistent with the results of carotid pathologic changes. These results confirmed that in addition to the effect of hyperglycemia on the artery in diabetic patients, the high level of inflammatory factors was also responsible for the pathological progression of blood vessels. A considerable contribution of the local inflammation of periodontitis to the inflammation level of the systemic system was also prominent.

As explored by Philip M. Preshaw et al. [26], diabetes combined with periodontitis group manifested a significantly increase in systemic inflammatory factors (IL-6, hs-CRP, TNF-α, IL-1β, IFN-γ), and periodontal treatment imparts a vital role in reducing systemic inflammation, which was in accordance with our findings. Our results obtained a negative correlation between serum adiponectin and hs-CRP levels, which signified that the treatment of periodontitis could reduce the levels of systemic inflammatory factors [26], increase serum adiponectin levels [35], delay the progression of diabetic insulin resistance and diabetic cardio-cerebrovascular complications. Furthermore, the anti-inflammatory property of adiponectin restricts the production of LPS-induced pro-inflammatory factors [18] and also reduces LPS-induced apoptosis [36].

As evident from our study, the progression of atherosclerosis is closely associated with both serum adiponectin and hs-CRP, but the balance of anti-inflammatory and pro-inflammatory factors are found to be more responsive to the inflammatory state of the system. Adamopoulos, Stamatis et al. [37] claimed the contribution of the balance of pro-inflammatory and anti-inflammatory cytokines to the improvement of left ventricular systolic function in dilated cardiomyopathy. The results confirmed that growth hormone-mediated effective regulation of the circulating cytokine network and soluble adhesion molecules in DCM patients, at the same time, strengthen the contraction reserve and reduce left ventricular volume. Wu Min et al. study revealed that compared with stable angina, higher levels of inflammatory factors and lower levels of anti-inflammatory factors were observed in patients with unstable angina and the change of pro-inflammatory/anti-inflammatory balance may represent the severity of coronary heart disease. In our study, a gradual reduction in the ratio of APN/hs-CRP was noted with the progression of the disease, compared to the A group, indicating deregulation of the balance of anti-inflammatory/pro-inflammatory with the advancement of the disease, especially in the compound group. Moreover, the arterial lesions in the compound group were more serious, suggesting that the ratio of APN/hs-CRP was consistent with the severity of the arterial lesions.

Atherosclerosis is a lipid-driven inflammatory disease of the lining of the arteries and the final clinical outcome depends on the balance of pro-inflammatory and inflammatory mitigation mechanisms. A large reduction in plasma LDL levels can lead to even severe atherosclerosis; plaque regression also minimizes the incidence of atherosclerotic cardiovascular disease [38]. Even though our results failed to obtain any statistically significant difference in TC, LDL, and TG between the C group and A group (*p* < 0.05), the HDL of the C group was significantly lower than that of the A group (*p* < 0.05). However, it is not completely consistent with the research results of Franca et al. [39], Nepomuceno et al. [40]. Franca's study reported significantly higher TC in the periodontitis group than in the normal control group. However, the diabetic periodontitis group demonstrated significantly elevated TC, LDL, and TG levels, while HDL, an anti-inflammatory lipoprotein, the combination of diabetes with periodontitis somehow prevented the HDL from significant decrease. In one prospective study of women, Small LDL and small HDLwere positively associated with diabetes, By contrast, large LDL and large HDL were inversely associated [41]. So HDL may inhibit development of T2DM by attenuating endoplasmic reticulum (ER) stress and apoptotic loss of pancreatic β-cells, an effect due in part to ABC transporter-mediated efflux of specific oxysterols with downstream activation of the hedghehog signalling receptor, Smoothened. The apoM-sphingosine-1-phosphate complex is critical to HDL antidiabetic activity, encompassing protection against insulin resistance, promotion of insulin secretion, enhanced β-cell survival and inhibition of hepatic glucose production [42]. Nepomuceno, R's meta-analysis results validated the significant correlation of PD with a decrease of HDL and an increase of LDL and triglyceride concentrations [40]; Only total serum HDL was measured in our experiment, It doesn't distinguish between large and small particles. Therefore, there are no clear results as to whether HDL promotes or inhibits diabetes progression as measured. Our study established the reduced anti-inflammatory effect of APN in the experimental group, while the pro-inflammatory factor hs-CRP was increased, especially in the periodontitis group and diabetic combined periodontitis group, destabilizing the pro-inflammatory and anti-inflammatory balance. The excess pro-inflammatory factor prompted the formation and progression of arterial plaque, thereby enhancing the prevalence of cardiovascular and cerebrovascular diseases.

## Conclusion

Periodontitis is a common chronic inflammatory disease induced by periodontal pathogens. The local inflammation of periodontitis aggravates the systemic inflammatory factors. At the same time, significant reduction of the serum APN destabilizes the homeostasis of lipid metabolism, increasing TG, TC, and LDL levels, and reducing HDL levels, which, in turn, accelerated the formation of atherosclerotic plaques and escalated the incidence of cardiovascular and cerebrovascular diseases.

However, there is the fact that the current study assessed the mentioned variables in rats; other studies were on humans. Some of the differences between the results could be attributed to this fact. In addition, there are some other limits. We only illustrated the pathological changes of the carotid artery in the natural course of periodontitis, lacking the inflammatory markers and Carotid pathologic changes after periodontitis intervention, which will be studied in the next animal experiment.

## Supplementary Information

**Additional file 1.** ARRIVE Guideline.

## Data Availability

All data generated or analysed during this study are included in this published article.
