# Supplementary material for: The effect of APN, hs-CRP and APN/hs-CRP in periodontitis with DAA
Source: BMC Oral Health. 2023 Feb 10;23:85. doi: 10.1186/s12903-023-02765-x (PMC9921664; doi:10.1186/s12903-023-02765-x)
Supplement: Supplementary file 1 — Additional file 1. ARRIVE Guideline. [file 12903_2023_2765_MOESM1_ESM.docx]

1. Study design：


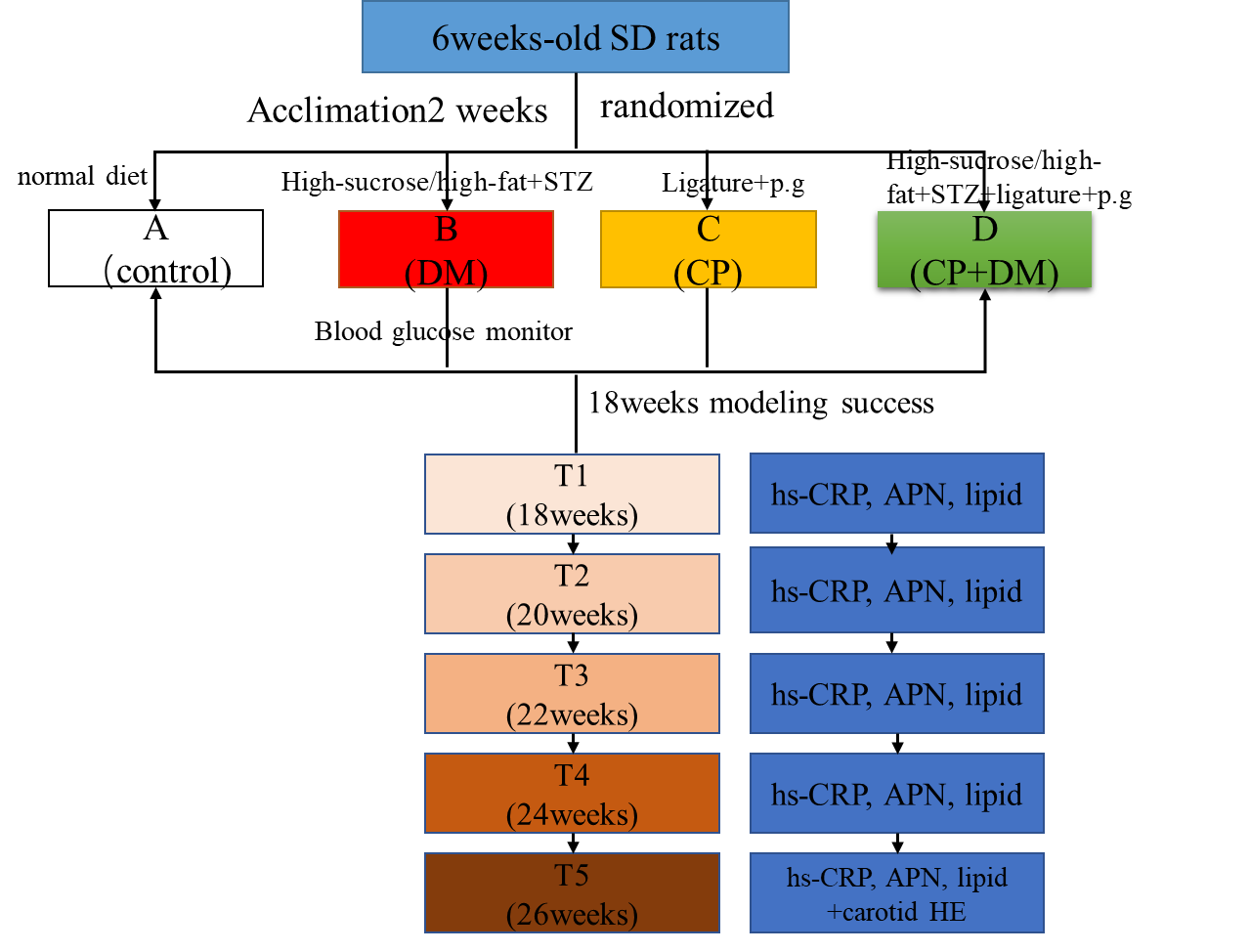
2.sample size：

Twenty-eight rats were randomly divided into the four groups， Sample size per group is (n=7).

3. The animals were included in the study after the successful establishment of models of chronic periodontitis and diabetes.

*4.* Twenty-eight 6-week-old SD male rats weighing *180 g (200 g) were purchased* from the animal center of Shanxi medical university（Taiyuan，China）and randomly divided into four groups (7rats/group): [normal](javascript:;) [control](javascript:;) [group](javascript:;)， Chronic periodontitis group，Diabetes group，Chronic periodontitis +Diabetes group.

5. For each animal, four different investigators were involved as follows: a first investigator administered the treatment based on the randomization table. This investigator was the only person aware of the treatment group allocation. A second investigator was responsible for the anaesthetic procedure, whereas a third investigator performed the Intraperitoneal injection of drugs and blood drawing. Finally, a fourth investigator (also unaware of treatment) assessed experimental data.

6.outcome measures

“The following parameters were assessed: Hs-CRP (high sensitivity C reactive protein); APN (diponectin), APN/hs-CRP, TG (triglyceride), TC (total cholesterol), LDL (low density lipoprotein), HDL (high-density lipoprotein), carotid HE staining.

7.All the data were represented by the figures. Data were presented as mean ±SD. SPSS software (version 21) was adopted for the statistical analysis. To determine the significance, one-way ANOVA with Tukey’s post-hoc test was employed for multi-group comparisons. Statistical significance was set at p < 0.05.

8. twenty-eight 6week male SD rats were used ,weighing 180 ~200 g, were purchased from the animal center of Shanxi medical university（Taiyuan，China）

9.

During diabetes modeling, caudal vein blood glucose was monitored at 13,14,15 and 16 weeks. Blood samples were collected at T1, T2, T3, T4, and T5 to detect hS-CRP, APN, and lipid.

These SD rats were fed in the Experimental Animal Center of Shanxi Medical University.

10.Result：

Result1：Establishment of T2DM model and the loss of alveolar bone

Figure1：fasting plasma glucose or random blood glucose was monitored. Model standard: FPG＞7.8 mmol/L, or PBG≥17.8 mmol/L.

Figure2 Bone resorption values of alveolar bone in each group. The CEJ value of alveolar bone in group B,C and D was significantly increased

A：normal control；B：DM；C：chronic periodontitis; D: DM+CP, compared to group A，**p*＜0.05.

Result2：Effect of adiponectin, hs-CRP on periodontitis with or without type 2 diabetes in rats

Table1，Figure 3：Disease progression documented a gradual reduction in the level of APN in groups B, C, and D. Serum adiponectin levels in group B decreased gradually with the progression of the disease, though the difference was statistically insignificant (p＞0.05). Compared to the A group, the significantly higher (p＜0.05).

Table2，Figure 4：The inflammatory factor hs-CRP in natural process was higher in groups B, C, and D, relative to group A. Serum hs-CRP gradually increased with the natural progression of the disease, but group B manifested no statistical difference (*p*＞0.05).

Result3：The ratio of APN/hs-CRP in each group

Table3，Figure5：The ratio of APN/hs-CRP signified the balance between anti-inflammation and pro-inflammation. The results revealed, at T1, significantly lower (p＜0.05) ratio of APN/CRP in D group, but no significant difference (p＞0.05) in group B, C group as compared to A group; at T2, compared to A group, the ratio was significantly lower (p＜0.05) in both B and D group. However, C group demonstrated no difference (p＞0.05); the ratio was significantly lower (p＜0.05) in the B, C, D group at T3, T4, T5, compared to A group. Overall, with the disease progression, the ratio of the B group failed to reflect any significant downward trend, whereas, C, D group manifested a gradual downward trend.

Result4：Serum lipid profile analysis.

Figure6：Serum lipid profile analysis. Compared to the A group, TG of group B, TC, LDL, TG of group D were significantly increased (p＜0.05). In the case of the C group, a significant decrease (p＜0.05) was obtained for HDL as compared to the A group. However, the other lipid indicators showed no significant difference (p＞0.05).

Result5：HE staining results of carotid arteries in each group

Figure 7：HE staining results of carotid arteries in each group. A group: An intact intima, flattened endothelial cells, orderly arrangement of the elastic fibers and smooth muscle cells of the media, no thickening of the vessel wall were observed; B group: Variation was noted in the blood vessel walls thickness, part of the endothelial cells were missing, the elastic fibers of the media were disordered, partially dissolved and fractured, and the smooth muscle cells were vacuolated; C group: The intima was incomplete, and some endothelial cells were exfoliated, the elastic fibers of the media were disordered, some of them were broken, and some specimens manifested calcium salt deposition; D group: The intima was incomplete, and some endothelial cells were missing, local necrosis of the smooth muscle tissue of the media was prominent, with amorphous particles and deepened staining, the elastic fibers in the necrotic area were disordered and some of them were broken
